# Supplementary material for: From sequence to enzyme mechanism using multi-label machine learning
Source: BMC Bioinformatics. 2014 May 19;15:150. doi: 10.1186/1471-2105-15-150 (PMC4229970; doi:10.1186/1471-2105-15-150)
Supplement: Additional file 2 — Java code of ml2db. Additional file ml2db_code.tar.gz contains the Java source code to run the multi-label machine learning experiments and save the results to database. The code’s Javadoc is included. [file 1471-2105-15-150-S2.zip › additional file 2/ml2db/utils/doc/uk/ac/standrews/utils/main/maputils/class-use/MapOfMaps.html]

Uses of Class uk.ac.standrews.utils.main.maputils.MapOfMaps


JavaScript is disabled on your browser.


- Overview
- Package
- Class
- Use
- Tree
- Deprecated
- Index
- Help

- Prev
- Next

- Frames
- No Frames

- All Classes

## Uses of Class uk.ac.standrews.utils.main.maputils.MapOfMaps

- Packages that use MapOfMaps

  | Package | Description |
  | --- | --- |
  |  |  |
  | --- | --- |
  | uk.ac.standrews.utils.main.database |  |
  | uk.ac.standrews.utils.main.maputils |  |
  | uk.ac.standrews.utils.test.maputils |  |
- - ### Uses of MapOfMaps in uk.ac.standrews.utils.main.database

    Methods in uk.ac.standrews.utils.main.database that return MapOfMaps

    | Modifier and Type | Method and Description |
    | --- | --- |
    |  |  |
    | --- | --- |
    | `MapOfMaps` | DbReader.`getColumnsValues(java.sql.ResultSet resultSet, int columnIndexOfExternalKeys, int columnIndexOfInternalKeys, int columnIndexOfValues, int expectedExternalKeysUniqueStrings, int expectedInternalKeysUniqueStrings, int expectedValuesUniqueStrings, java.lang.String defaultValue, boolean acceptEmptyStrings, java.lang.String prefix_for_column_2)` Get three columns of a `ResultSet` and store them in a [external key, internal key, value] map |
    | `MapOfMaps` | DbReader.`getMap(java.lang.String sql, int uniqueStrings, java.lang.String defaultValue, boolean acceptEmptyStrings, java.lang.String prefix_for_column_2)` Converts a query resultset into a map. |
  - ### Uses of MapOfMaps in uk.ac.standrews.utils.main.maputils

    Methods in uk.ac.standrews.utils.main.maputils with parameters of type MapOfMaps

    | Modifier and Type | Method and Description |
    | --- | --- |
    |  |  |
    | --- | --- |
    | `void` | MapOfMaps.`addAll(MapOfMaps anotherMap)` |
  - ### Uses of MapOfMaps in uk.ac.standrews.utils.test.maputils

    Methods in uk.ac.standrews.utils.test.maputils that return MapOfMaps

    | Modifier and Type | Method and Description |
    | --- | --- |
    |  |  |
    | --- | --- |
    | `static MapOfMaps` | MapOfMapsTest.`getMap1(boolean acceptsEmptyStrings)` |

- Overview
- Package
- Class
- Use
- Tree
- Deprecated
- Index
- Help

- Prev
- Next

- Frames
- No Frames

- All Classes
